# Optimized deep learning-accelerated single-breath-hold abdominal HASTE with and without fat saturation improves and accelerates abdominal imaging at 3 Tesla

**DOI:** 10.1186/s12880-025-01838-3

**Published:** 2025-09-18

**Authors:** Qinxuan Tan, Felix Kubicka, Dominik Nickel, Elisabeth Weiland, Bernd Hamm, Dominik Geisel, Moritz Wagner, Thula C. Walter-Rittel

**Affiliations:** 1 Department of Radiology, Charité - Universitätsmedizin Berlin, Freie Universität Berlin, Humboldt-Universität zu Berlin, and Berlin Institute of Health, Berlin, Germany; 2 MR Applications Predevelopment, Siemens Healthcare GmbH, Allee am Roethelheimpark 2, 91052 Erlangen, Germany

**Keywords:** Deep learning, Artificial intelligence, Magnetic resonance imaging, Abdomen

## Abstract

**Background:**

Deep learning-accelerated single-shot turbo-spin-echo techniques (DL-HASTE) enable single-breath-hold T2-weighted abdominal imaging. However, studies evaluating the image quality of DL-HASTE with and without fat saturation (FS) remain limited. This study aimed to prospectively evaluate the technical feasibility and image quality of abdominal DL-HASTE with and without FS at 3 Tesla.

**Materials and methods:**

DL-HASTE of the upper abdomen was acquired with variable sequence parameters regarding FS, flip angle (FA) and field of view (FOV) in 10 healthy volunteers and 50 patients. DL-HASTE sequences were compared to clinical sequences (HASTE, HASTE-FS and T2-TSE-FS BLADE). Two radiologists independently assessed the sequences regarding scores of overall image quality, delineation of abdominal organs, artifacts and fat saturation using a Likert scale (range: 1–5).

**Results:**

Breath-hold time of DL-HASTE and DL-HASTE-FS was 21 ± 2 s with fixed FA and 20 ± 2 s with variable FA (*p* < 0.001), with no overall image quality difference (*p* > 0.05). DL-HASTE required a 10% larger FOV than DL-HASTE-FS to avoid aliasing artifacts from subcutaneous fat. Both DL-HASTE and DL-HASTE-FS had significantly higher overall image quality scores than standard HASTE acquisitions (DL-HASTE vs. HASTE: 4.8 ± 0.40 vs. 4.1 ± 0.50; DL-HASTE-FS vs. HASTE-FS: 4.6 ± 0.50 vs. 3.6 ± 0.60; *p* < 0.001). Compared to the T2-TSE-FS BLADE, DL-HASTE-FS provided higher overall image quality (4.6 ± 0.50 vs. 4.3 ± 0.63, *p* = 0.011). DL-HASTE achieved significant higher image quality (*p* = 0.006) and higher sharpness score of organs compared to DL-HASTE-FS (*p* < 0.001).

**Conclusion:**

Deep learning-accelerated HASTE with and without fat saturation were both feasible at 3 Tesla and showed improved image quality compared to conventional sequences.

**Trial registration:**

Not applicable.

## Introduction

Magnetic resonance imaging (MRI) is a central diagnostic tool to assess abdominal pathology, due to its high soft-tissue contrast [1]. T2-weighted (T2w) imaging plays a critical role for anatomic assessment and lesion detection [2, 3]. Hence, upper abdominal imaging protocols should include motion resistant T2w sequences with and without fat saturation (FS) [2, 4–6].

In clinical routine, single shot turbo spin-echo (TSE) sequences (e.g., the half-Fourier acquisition single-shot turbo spin echo, HASTE) and T2-TSE-FS BLADE sequences (proprietary name for periodically rotated overlapping parallel lines with enhanced reconstruction technique) are generally considered to be the standard imaging sequences in abdominal imaging [2, 7]. For routine upper abdominal imaging, HASTE can be acquired as a multi-breath-hold acquisition and T2-TSE-FS BLADE either as multi-breath-hold or as navigator-triggered acquisition [7]. FS techniques should generally be used for at least one set of T2w images of the upper abdomen [6–8]. Images without FS are especially helpful for assessing organ anatomy, while FS provide improved visualization of pathologic lesions [2, 3].

Over the past decades, different innovative techniques have been proposed to accelerate T2w imaging, such as parallel imaging (PI), and, more recently, deep learning-based (DL) reconstructions [9, 10]. With convolutional neural networks (CNNs), DL reconstruction techniques allow higher PI acceleration factors while simultaneously maintaining comparable image quality [10, 11]. These algorithms can reconstruct high image quality from noisy, under sampled k-space data [12]. Previous studies have shown that DL-HASTE sequences enable single-breath-hold upper abdominal imaging with acquisition times of approximately 16–21 s, significantly reducing scan times compared to multi-breath-hold and navigator-triggered T2w sequences at both 1.5 Tesla and 3.0 Tesla [13–19]. Compared to DL-HASTE sequences, multi-breath-hold HASTE requires approximately 3–4 breath-holds (40–60 s), while navigator-triggered BLADE sequences typically take more than 120s in a clinical setting. In addition to fast acquisition times, DL-HASTE sequences also showed comparable or even improved image quality for abdominal MRI [13–19]. Nevertheless, these findings remain limited to specific applications, and further research is required to evaluate the performance of DL-HASTE across different anatomical regions and acquisition protocols [20].

At 3 Tesla, single-breath-hold HASTE may be limited by the specific absorption rate (SAR) due to its high number of refocusing pulses and long echo train lengths [21]. To solve this problem, the application of variable refocusing flip angle (FA) was suggested. Previous study showed that single-breath-hold DL-HASTE-FS with variable FA can reduce SAR in abdominal image at 3 Tesla [16, 22, 23].

Previous studies also demonstrated that DL-HASTE-FS provided promising results in detecting liver lesion with high diagnostic confidence [13–17]. More recently, several publications have also reported on the potential of DL-HASTE for the assessment of complex abdominal pathology, such as pancreatic lesions [18, 19]. However, to the best of our knowledge, no direct comparisons of DL-HASTE with fixed FA acquisitions have been conducted, nor have any studies evaluated the differences between DL-HASTE with and without FS directly.

The aim of our study was to evaluate abdominal DL-HASTE with and without FS at 3 Tesla regarding acquisition time, overall image quality, delineation of abdominal organs and presence of artifacts. To this end, we investigated how different sequence parameters (fixed FA vs. variable FA; different FOV size) affect image quality and compared DL-HASTE and DL-HASTE-FS to conventional T2-weightes sequences used in clinical routine (HASTE, HASTE-FS, and T2-TSE-FS BLADE).

## Materials and methods

### Patient study

This prospective study was approved by the institutional review board (Ethikkommission Charité Berlin [EA1/344/21]), informed consent was obtained from all volunteers and patients. The study group included of 10 volunteers and 52 consecutive patients who were referred to our department for upper abdominal MRI. Patients included in this study were referred for imaging to our department as part of routine clinical imaging.

### MR imaging parameters

**Fig. 1:**
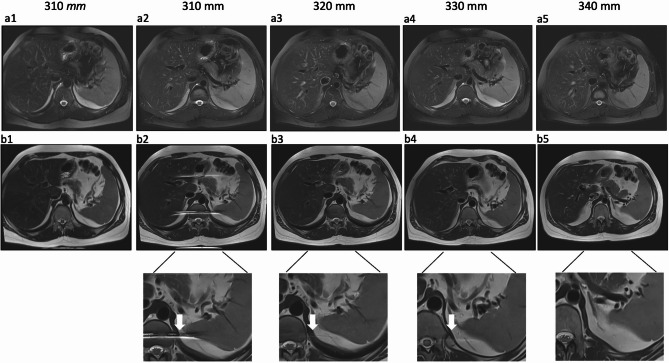
Effect of FOV size on image quality of DL-HASTE FS and DL-HASTE from one same volunteer (**a1**: HASTE-FS, **a2**-**a5**: DL-HASTE-FS; **b1**: standard HASTE, **b2**-**b5** DL-HASTE). The comparison was performed using a variable FA scheme set at 130–90–110–130°. To avoid fold-over type artifacts (arrows), the FOV needed to be about 10% larger in DL-HASTE compared to DL-HASTE-FS

All examinations were performed on a clinical 3 Tesla MRI system (MAGNETOM Vida; Siemens Healthineers, Erlangen, Germany) with patients in supine position using an 18-channel body/spine array coil. First, the effect of different FA and FOV size on DL-HASTE image quality was evaluated in 10 healthy volunteers to determine optimal DL parameters. Evaluated FA schemes included a fixed FA of 115° and a variable FA pattern of 130–90–110–130°. The variable FA scheme used here was a fixed pattern implemented by Siemens as part of the research prototype [22]. FOV values ranged from 310 mm to 340 mm in 10 mm increments. The FOV comparison results are presented in Fig. 1.

Secondly, using optimized parameters derived from the volunteer study, DL-HASTE sequences were acquired in all patients followed by conventional sequences (HASTE, HASTE-FS, BLADE-FS) in a fixed acquisition order. Phase oversampling was consistently set to 0% for both conventional and DL-HASTE sequences. For BLADE, the phase oversampling was set to 70%, which reflects the default vendor-recommended setting on our scanner. Detailed imaging parameters are displayed in Table 1.

**Table 1:** Acquisition parameters

Parameters	HASTE	HASTE-FS	BLADE-FS	DL-HASTE fixed FA	DL-HASTE variable FA	DL-HASTE- FS fixed FA	DL-HASTE-FS variable FA
Orientation	Axial	Axial	Axial	Axial	Axial	Axial	Axial
TA, min: s	0:45	0:45	04:39 − 10:10	0:20*	0:19*	0:20*	0:19*
TE/TR, ms	95/1100	95/1100	89/>3000	96/565*	96/535*	96/565*	96/535*
FA, degrees	160	160	100	115	130-90-110-130	115	130-90-110-130
iPAT	2	2	3	3	3	3	3
Turbo Factor	43	253	253	253	253	253	253
Matrix size	384 × 253	384 × 253	320 × 320	384 × 253	384 × 253	384 × 253	384 × 253
Motion management	3 BH	3 BH	Navigator triggering	1 BH	1 BH	1 BH	1 BH
Slice thickness, mm	5	5	5	5	5	5	5
Slice	35	35	39	35	35	35	35
FOV^◊^, mm^2^	308 × 380	308 × 380	380 × 380	308 × 380	308 × 380	308 × 380	308 × 380

HASTE, half-Fourier single shot turbo spin echo; FS, fat saturation; DL, deep learning reconstruction; FA, flip angle; TA, time of acquisition; TE/TR, echo time/repetition time; iPAT, integrated Parallel Acquisition Techniques; BH, breath-hold; FOV, field of view

*TR was individually adjusted to specific absorption rate (SAR) limits, ranging from 535ms to 753ms, which resulted in variable TA

◊ FOV in this table is the default value, which was individually adjusted in clinical

### HASTE with DL reconstruction

The proprietary Work-in-Progress (WIP) sequence used in our study was provided by Siemens Healthineers. The employed HASTE sequence with DL-based reconstruction is a research application using a modified sequence and reconstruction. The sequence acquires k-space data using a regular cartesian sampling as known from parallel imaging with a separate acquisition of the calibration data to estimate coil sensitivity maps. To reduce crosstalk in acquisitions with lower TR, the slice increment between consecutively acquired lines is increased to 4. Furthermore, a variable flip angle evolution is supported for the refocusing pulses in the echo train [21, 22]. The DL-based reconstruction is based on a variational network [24]. Compared to versions used in earlier works [13–17], the network architecture was modified and particularly the regularization component changed to a hierarchical design as first explored in the context of prostate imaging [25]. Similarly to the previously employed networks, the training was performed offline using about 10,000 slices obtained from volunteers with informed written consent and in accordance with local IRB guidelines and latter integrated into the reconstruction pipeline of the scanner for prospective use.

### Qualitative MRI evaluation

Image data was presented in random order to two blinded radiologists (with 4 and 15 years of clinical experience in abdominal MRI) who performed image analysis independently. The readers evaluated overall image quality and delineation of abdominal organs (overall sharpness score; organ-based sharpness score: liver: porta hepatis level, spleen: splenic hilum level, kidney: kidney hilum level, the entire pancreas), breathing related motion artifacts, and homogeneity of fat saturation. All of these categories were rated subjectively with an ordinal 5-point Likert scale (Table 2) [16].

**Table 2:** Scoring for various parameters of image quality for each sequence

	Score	Scoring system
Overall image quality	1–5	1. Unacceptable; 2. Poor; 3. Moderate; 4. Good; 5. Excellent
Overall sharpness score	1–5	1. Unacceptable; 2. Poor; 3. Moderate; 4. Good; 5. Excellent
Delineation (sharpness) of abdominal organs (Liver, Spleen, Kidney, Pancreas and Bowel)	1–5	1. Unacceptable; 2. Poor; 3. Moderate; 4. Good; 5. Excellent
Breath-hold related motion artifacts	1–5	1. Unacceptable; 2. Extreme blur; 3. Moderate blurring; 4. Mild blurring; 5. No blurring
Homogeneity of fat suppression	1–5	1. Unacceptable; 2. Poor; 3. Acceptable; 4. Good; 5. Excellent

### Statistical analysis

All statistical analyses was performed using SPSS version 29 (IBM Corp, Armonk, NY). The mean image quality scores determined by two observers for each quality metric were tabulated. The sample size estimation was based on a paired design with a two-sided superiority hypothesis. Assuming a medium effect size (Cohen’s d ≈ 0.6) for pairwise differences in visual scores [26], a range of 40–60 subjects would be sufficient to achieve 80% power at a significance level of 0.05. Then, we applied the paired Wilcoxon signed-rank test to compare the acquisitions for image quality between DL and clinical routine sequences. Continuous variables were compared using the paired sample t test (or the Wilcoxon test, in the case of a skewed distribution). P values less than 0.05 were considered to indicate a significant difference. For each sequence, we evaluated consistency in reader scores on all assessed parameters using intra-class correlation coefficient (ICC) analysis, where < 0.4 = poor agreement; 0.4–0.59 = fair agreement; 0.6–0.74 = good agreement; and 0.75-1 = excellent agreement.

## Results

### Patients

Of the 52 included patients, two were excluded, one due to technical reasons and the other due to severe ascites resulting in poor breath-hold compliance and severe artifacts in all MR sequences. 50 patients were included in the study (31 males and 29 females, age ranging from 24 to 83 years, with a mean age of 63.2 ± 19.6 years). In these patients, lesions were located in the liver (*n* = 6), pancreas (*n* = 6), kidney (*n* = 18), or more than one of these organs (*n* = 20), reflecting the common spectrum of upper abdominal pathologies.

### MR imaging parameters

In initial volunteer examinations (*n* = 10), DL-HASTE without FS demonstrated aliasing when used with a standard FOV (308 × 379 mm^2^) (Fig. 1, b2–4, arrow), but DL-HASTE-FS did not (Fig. 1, a2). To systematically evaluate the impact of FOV on image quality, we evaluated the effect of an increasing FOV size on DL-HASTE and DL-HASTE-FS. Our findings indicate that to avoid aliasing artifacts in DL-HASTE, FOV sizing should be about 10% larger in DL-HASTE compared to DL-HASTE-FS (Fig. 1) and standard HASTE sequences. Hence, FOV size settings were increased in subsequent imaging.

Secondly, we evaluated the impact of different FA on both acquisition time and image quality in healthy volunteers. Our findings showed that mean acquisition time of DL-HASTE and DL-HASTE-FS with fixed FA were both 21 ± 2s (DL-HASTE range 20–23 s; TR 565-657ms; DL-HASTE-FS: range 20–26 s; TR 566-753ms). Mean acquisition time of DL-HASTE and DL-HASTE-FS with variable FA were both 20 ± 2s (DL-HASTE: range 19–24 s; TR 535-686ms; DL-HASTE-FS: range 19–25 s; TR 536-708ms). Overall, using a variable FA reduced the acquisition time of DL-HASTE by 1.0s (range 1–2 s; *p* < 0.001), and DL-HASTE-FS by 1.1s (range 1–2 s; *p* < 0.001).

No significant differences were observed for image quality for DL-HASTE with and without FS using fixed vs. variable FA (DL-HASTE fixed FA: 4.8 ± 0.39 vs. variable FA: 4.8 ± 0.40, *p* = 0.317; DL-HASTE-FS fixed FA: 4.6 ± 0.49 vs. variable FA: 4.6 ± 0.50, *p* = 0.564). Hence, only the variable FA-setting was used in the evaluation of image quality, since a variable FA provided shorter acquisition times and improved image quality, albeit not significantly so.

The optimized acquisition times for the optimized DL-HASTE with and without FS were significantly shorter (both 21 ± 2s: *p* < 0.001) compared to HASTE and HASTE-FS (65s; 3 breath-hold acquisitions of 15s and 2 × 10s pauses) and T2-TSE-FS BLADE with respiratory triggering (04:39 − 10:10 min; mean 6:43 ± 1:45 min).

### Inter-observer agreement

Inter-class correlation coefficient (ICC) between two readers for the all assessed parameters was fair for HASTE-FS and DL-HASTE-FS (HASTE-FS: 0.52; range 0.38–0.63; DL-HASTE-FS: 0.53; range 0.39–0.64), fair to excellent for HASTE and DL-HASTE (HASTE: 0.40; range 0.20–0.55; DL-HASTE: 0.80; range 0.56–0.91), and good for T2-TSE-FS BLADE (ICC: 0.74; range 0.67–0.80). In the following text, only the mean image quality scores from two observers are provided.

### Qualitative MRI evaluation

**Fig. 2:**
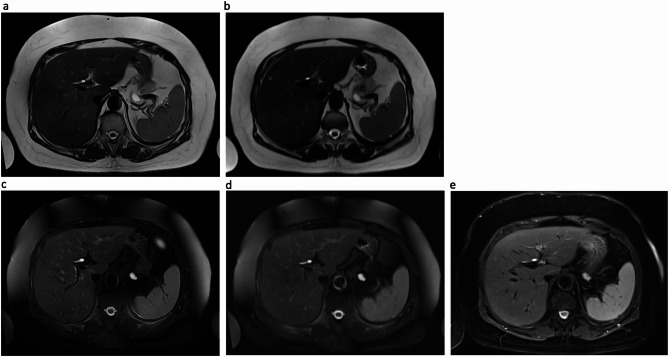
A 75-year-old woman with a pancreatic lesion. Comparing overall image quality of DL-HASTE (**a**) and DL-HASTE-FS (**c**) with standard multi-breath hold HASTE (**b**) and HASTE-FS (**d**) as well as navigator triggered T2-TSE-FS BLADE (**e**). Image quality was rated highest for DL-HASTE (**a**) and DL-HASTE-FS (**c**). In Fig. 2**c** and **d**, the dark band-like signal loss observed in the subcutaneous fat likely results from inhomogeneous fat suppression using the SPAIR technique, which can be affected by B₀ field non-uniformity in the upper abdominal region

Both DL-HASTE and DL-HASTE-FS had significantly higher overall image quality scores compared to standard multi-breath-hold HASTE sequences (4.8 ± 0.40 vs. 4.1 ± 0.50 and 4.6 ± 0.50 vs. 3.6 ± 0.60; *p* < 0.001; Fig. 2). DL-HASTE-FS also provided higher overall image quality compared to T2-TSE-FS BLADE (4.6 ± 0.50 vs. 4.3 ± 0.63, *p* = 0.011; Fig. 2).

**Fig. 3:**
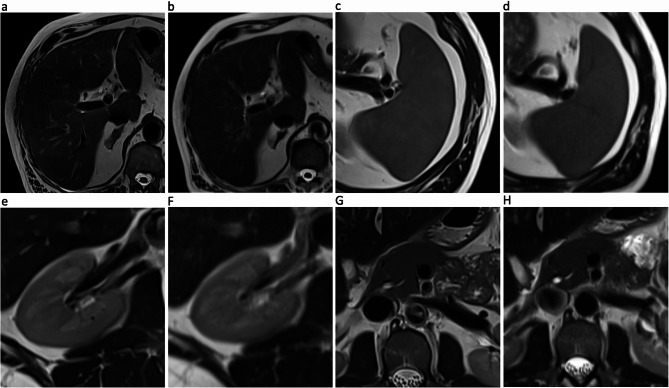
Sharpness of organ margins with DL-HASTE (**a**, **c**, **e**, **g**) compared to HASTE (**b**, **d**, **f**, **h**). DL-HASTE received the highest scores for all upper abdominal organ margins. All images were acquired in the same patient

**Fig. 4:**
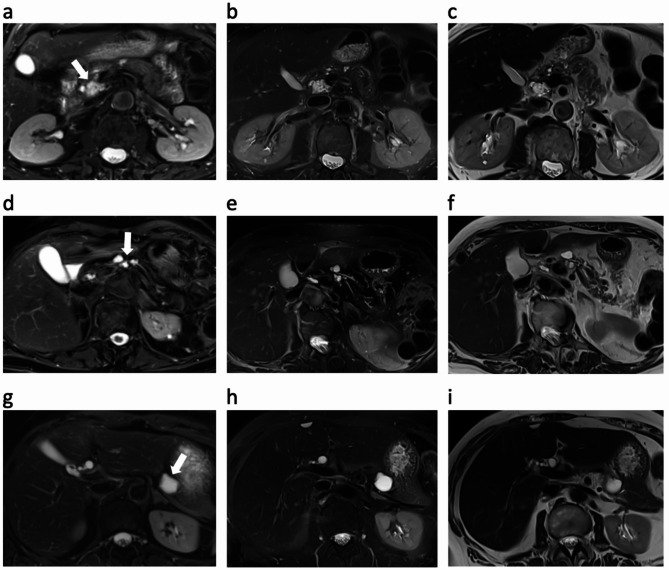
Comparison of organ margin delineation in T2-TSE-FS BLADE (**a**, **d**, **g**) with DL-HASTE-FS (**b**, **e**, **h**), and DL-HASTE (**c**, **f**, **i**) in three patients with cystic pancreatic lesions (arrows) in the head (**a**-**c**), body (**d**-**f**), and tail (**g**-**i**). Note the optimal visualization of pancreatic anatomy with DL-HASTE

DL-HASTE and DL-HASTE-FS demonstrated significantly higher scores for sharpness of abdominal organs compared to HASTE and HASTE-FS (Tables 3 and 4). DL-HASTE-FS also outperformed T2-TSE-FS BLADE with higher scores of sharpness, especially from kidney (4.8 ± 0.40 vs. 4.6 ± 0.54; *p* = 0.005), pancreas (4.1 ± 0.69 vs. 3.1 ± 0.52; *p* < 0.001) and bowel (4.4 ± 0.50 vs. 3.5 ± 0.54; *p* < 0.001) (Fig. 3). Overall, anatomic margin of organs was best delineated with DL-HASTE (highest overall sharpness score: 4.9 ± 0.38; *p* < 0.001) (Figs. 3 and 4; Tables 3 and 4).

**Table 3:** Image quality scores of DL sequences (DL-HASTE, DL-HASTE-FS) with clinical standard sequences (HASTE, HASTE-FS, BLADE FS)

	HASTE	DL-HASTE variable FA	HASTE-FS	BLADE-FS	DL-HASTE-FS variable FA
Overall Image Quality	4.1 ± 0.50	4.8 ± 0.40	3.6 ± 0.60	4.3 ± 0.63	4.6 ± 0.50
Overall sharpness	4.2 ± 0.55	4.9 ± 0.38	3.5 ± 0.67	4.0 ± 0.73	4.4 ± 0.62
Sharpness of margin (Liver)	4.2 ± 0.60	5.0 ± 0.19	3.6 ± 0.62	4.2 ± 0.58	4.3 ± 0.51
Sharpness of margin (Spleen)	4.2 ± 0.72	4.7 ± 0.59	3.5 ± 0.83	4.3 ± 0.69	4.1 ± 0.67
Sharpness of margin (Kidney)	4.3 ± 0.49	5.0 ± 0.23	3.9 ± 0.59	4.6 ± 0.54	4.8 ± 0.40
Sharpness of margin (Pancreas)	4.2 ± 0.49	4.8 ± 0.40	3.6 ± 0.63	3.1 ± 0.52	4.1 ± 0.69
Sharpness of margin (Bowel)	4.1 ± 0.40	4.9 ± 0.31	3.2 ± 0.50	3.5 ± 0.54	4.4 ± 0.50
Motion Artifact	4.4 ± 0.77	4.6 ± 0.57	4.3 ± 0.76	4.9 ± 0.44	4.5 ± 0.60
Fat Saturation	-	-	3.4 ± 0.68	4.7 ± 0.45	3.5 ± 0.57

**Table 4:** P value for comparing DL sequences (DL-HASTE, DL-HASTE-FS) with clinical standard sequences (HASTE, HASTE-FS, BLADE FS)

	HASTE vs. DL-HASTE	HASTE-FS vs. DL-HASTE-FS	BLADE-FS vs. DL-HASTE-FS	DL-HASTE vs. DL-HASTE-FS
Overall Image Quality	< 0.001	< 0.001	0.011	0.006
Overall sharpness	< 0.001	< 0.001	< 0.001	< 0.001
Sharpness of margin (Liver)	< 0.001	< 0.001	0.061	< 0.001
Sharpness of margin (Spleen)	< 0.001	< 0.001	0.221	< 0.001
Sharpness of margin (Kidney)	< 0.001	< 0.001	0.005	0.005
Sharpness of margin (Pancreas)	< 0.001	< 0.001	< 0.001	< 0.001
Sharpness of margin (Bowel)	< 0.001	0.012	< 0.001	< 0.001
Motion Artifacts	0.012	0.018	< 0.001	0.564
Fat Saturation	-	0.157	< 0.001	-

Regarding breathing artifacts, DL-HASTE and DL-HASTE-FS showed significantly better scores compared to conventional multi-breath-hold HASTE (DL-HASTE vs. HASTE: 4.6 ± 0.57 vs. 4.4 ± 0.77: *p* = 0.012; DL-HASTE-FS vs. HASTE-FS: 4.5 ± 0.60 vs. 4.3 ± 0.76: *p* = 0.018), while navigator-trigger T2-TSE-FS BLADE received the highest scores (4.9 ± 0.44) (Tables 3 and 4). DL-HASTE-FS and HASTE-FS both received suboptimal scores for homogeneity of fat saturation compared to T2-TSE-FS BLADE (3.5 ± 0.57 vs. 3.4 ± 0.68 vs. 4.7 ± 0.45, *p* < 0.001). (Fig. 1; Tables 3 and 4).

Our findings also showed that DL-HASTE achieved significantly superior overall image quality compared to DL-HASTE-FS (*p* = 0.006). Compared to DL-HASTE, DL-HASTE-FS received significantly lower scores for delineation of organ margins due to suboptimal fat saturation (*p* < 0.001).

## Discussion

This prospective study investigated technical feasibility and clinical performance of optimized DL-HASTE with and without FS in upper abdominal imaging at 3 Tesla. Our results demonstrated that DL-HASTE and DL-HASTE-FS enabled a significant reduction of the acquisition time and achieved improved overall image quality compared to clinical routine sequences. The present study found that a 10% larger FOV was necessary for DL-HASTE compared to DL-HASTE-FS to avoid aliasing artifacts, and both sequences were best performed with variable FA. More importantly, our study demonstrated the high image quality and robustness of DL-HASTE without FS.

Increased scan times are a key limitation of MR imaging [10, 27]. Over the years, significant improvements have been made to accelerate imaging. These advancements are owed to a combination of advances and improvements in MR hardware and innovations in both image acquisition and image reconstruction strategies [9, 10]. Parallel imaging (PI) techniques such as SENSE (sensitivity encoding) and GRAPPA (generalized auto calibrating partially parallel acquisitions) are established in clinical MRI [25]. Although GRAPPA is routinely used in clinical practice, image quality at high acceleration factors (e.g. R *≥* 3) can be degraded by noise amplification and/or under-sampling aliasing artifacts [25, 27]. Previous studies have shown that DL-HASTE sequences allow for single-breath-hold upper abdominal imaging, with an average acquisition time of 16–21 s, which significantly reduces scan time compared to multi-breath-hold and navigator-triggered acquisitions at 1.5 T and 3.0T [13–19]. These findings are in line with the findings in our study. Compared to conventional T2-HASTE with acceleration factor (iPat) 2, we used iPat 3 in DL-HASTE sequences to reduce acquisition time. In DL-HASTE, acquisition time depends on the repetition time (TE) and the number of slices, but the former is affected by SAR limitations. As a result, acquisition time varies between patients in our present study (range 19–25 s).

At 3 Tesla MRI, DL-HASTE sequences achieved higher signal-to-noise ratios (SNR) and reduced motion artifacts, improving image sharpness of organ delineation [16, 18, 19]. However, for 3.0 Tesla, significant limitations for the acquisition time of DL-HASTE sequence are imposed by SAR constraints. To solve this limitation, the application of variable refocusing FAs was suggested in previous studies [20, 28]. Of the various DL-HASTE studies at 3T [15, 16, 18, 19, 22, 23], more than half adopted variable FA approaches to reduce SAR limitations and preserve image quality. For example, Herrmann et al. [15, 22] used a 130-90-110-130° scheme, Ichinohe et al. [23] applied 130-60-100-45°, and Wary et al. used 130-110-120-140° [16]. Herrmann et al. explored five different variable FA with the goal of determining the optimal FA, providing low SAR and acceptable image quality. Their results showed that variable FAs of 130-90-110-130° was optimal, as it provided the best balance between lower SAR, reduced cardiac-motion-related signal loss and improved image quality [21]. Compared with conventional reconstruction, DL reconstruction significantly enhanced the image quality and therefore allowed the use of lower FAs. In the study presented here, DL-HASTE image with fixed flip angle (115°) and variable FA (130-90-110-130°) were compared as described by Herrmann et al. [21]. Our results showed that the use of a variable FA shorted the acquisition time of DL-HASTE by 1.1s (range 1–2 s, *p* < 0.001) without negatively affecting image quality. Hence, overall, our study supports the use of variable FA at 3 Tesla.

In our study, DL-HASTE-FS achieved significantly superior image quality and sharpness of abdominal organs compared to conventional HASTE-FS. The results of our study agree with previously reported data [14, 15, 19]. However, when comparing DL-HASTE-FS with the BLADE sequence, previous studies have reported controversial findings [13–17, 22]. Herrmann et al. [14, 15, 22] reported that BLADE achieved higher overall image quality scores than DL-HASTE-FS on both 1.5 and 3.0T MRI. Additionally, BLADE and DL-HASTE-FS showed similar performance in delineating the margins of upper abdominal organs (liver, pancreas, spleen and kidneys) without significant difference [15]. However, Shanbhogue et al. [13] and Wary et al. [16] reported that the DL-HASTE-FS sequence improved image quality and liver contrast compared to BLADE. In the study by Mulé et al. [17], DL-HASTE-FS demonstrated higher overall quality compared to BLADE but showed a lower median noise level in the liver. Our results were consistent with the findings of Shanbhogue et al. and Wary et al., and we found that DL-HASTE achieved clearer delineation of margins of abdominal organs. The reasons for these controversial findings may be attributed to the following factors. Firstly, Herrmann et al. [15] demonstrated lower overall image quality scores for DL-HASTE-FS compared to BLADE, but organ-based assessments showed no significant differences for liver, kidney, pancreas, and spleen, whereas our study evaluated the overall image quality of the upper abdomen. Secondly, we found that BLADE-FS demonstrated blurred margins in regions such as the pancreas and bowel, which may be attributable to the longer acquisition times and motion averaging inherent to BLADE [19]. Thirdly, the protocols and DL-reconstruction methods described in the presented study here vary somewhat from those described in previous studies [13–19, 22]. And finally, differences in fat suppression uniformity were observed among sequences in our study, with BLADE performing best, followed by DL-HASTE-FS and then HASTE-FS, likely due to the limitations of SPAIR at 3T due to B₀ inhomogeneities [29].

From a diagnostic perspective, previous studies have shown that DL-HASTE-FS achieved comparable performance to conventional T2-weighted sequences in detecting and measuring hepatic lesions, with significantly better conspicuity for small lesions [13, 14, 16, 17]. Wary et al. [16] reported that 71% of small focal hepatic lesions were rated with good to excellent conspicuity on DL-HASTE-FS, compared to only 39% on BLADE. Regarding DL-HASTE without fat suppression, Liu et al. [19] demonstrated excellent lesion conspicuity and contrast in pancreatic imaging, improving radiologist confidence. Similarly, Kim et al. [18] found that conventional HASTE sequences missed small cystic pancreatic lesions in approximately 31% of patients when compared to DL-HASTE. Additionally, across these studies, inter-reader agreement was consistently high. These findings suggest that the enhanced image quality of DL-HASTE sequences may contribute to more confident and consistent diagnoses, particularly in anatomically complex regions.

To our knowledge, no previous studies have directly compared DL-HASTE-FS and DL-HASTE. In our study, DL-HASTE showed superior performance in overall image quality and organ delineation, particularly in anatomically complex regions. These findings suggest that DL-HASTE may be better suited for evaluating anatomical detail, while DL-HASTE-FS has demonstrated benefits in detecting solid lesions, as supported by previous studies [16]. Understanding the advantages of each sequence may be helpful for optimizing imaging protocols and considering their use in follow-up or abbreviated MRI workflows s in the future.

Our study has some limitations. The main limitation of our study is that we did not focus on a specific pathology but rather performed a general evaluation of the image quality of upper abdominal organs. However, in our study we highlight the particular benefit of DL-HASTE without FS in depicting upper abdominal anatomy, which is generally helpful in diagnosing abdominal pathologies, especially in complex areas such as pancreatic region. Secondly, a relatively small number of patients was included. However, all patients underwent an identical DL-HASTE protocol and significant differences were still detected in most measurements. Thirdly, image quality was evaluated qualitatively in our study, as previously published studies revealed that quantitative measurement method such SNR or contrast-to-noise ratio (CNR) may be affected in parallel imaging, as noise is not evenly distributed in accelerated images reconstructed with parallel technique [30, 31]. Moreover, our study only evaluated DL-HASTE with and without FS on a single-vendor 3T system. Specific parameters and sequence performance may vary across vendors. Finally, a detailed analysis of SAR-variations between the sequences and parameter settings was not performed. Instead, our observations regarding SAR were based on the scanner’s automatic TR adjustments during DL-HASTE acquisitions. In the clinically evaluated the variable FA scheme, TR values were generally shorter, and no SAR warnings were observed for the evaluated DL sequences, hence a detailed SAR evaluation was not part of this study.

In conclusion, this study demonstrated that DL-accelerated HASTE sequences, both with and without FS, provide high-quality abdominal imaging at 3 Tesla with a single breath-hold. Compared to conventional multi-breath-hold HASTE and navigator-triggered T2-TSE-FS BLADE sequences, DL-HASTE techniques achieved superior overall image quality, improved sharpness of abdominal organ delineation, and reduced acquisition times. Notably, DL-HASTE without FS exhibited the highest scores for image quality and anatomical detail, particularly in complex regions such as the pancreas. The implementation of variable flip angles further optimized imaging by reducing acquisition times without compromising quality. These findings further highlight the efficacy of DL-HASTE and DL-HASTE-FS in abdominal imaging and underscore their future role in clinical routine.

## Data Availability

The datasets generated and analysed during the current study are not publicly available due to data protection and privacy regulations but are available from the corresponding author on reasonable request and with appropriate institutional approvals.
